# Elevated Troponin I as a Marker for Unfavorable Outcomes in Acute Ischemic Stroke

**DOI:** 10.7759/cureus.49568

**Published:** 2023-11-28

**Authors:** Tareq Esteak, Mashfiqul Hasan, Mohammad Atiqur Rahman, Dewan Mohammad Karimul Islam, Swapan Kumar Ray, Ahmed Hosain, Sarah Alam, Tahira Zannat, ATM Hasibul Hasan, Sharif Uddin Khan

**Affiliations:** 1 Clinical Neurology, National Institute of Neurosciences and Hospital, Dhaka, BGD; 2 Endocrinology and Metabolism, Bangabandhu Sheikh Mujib Medical University, Dhaka, BGD; 3 Endocrinology and Metabolism, National Institute of Neurosciences and Hospital, Dhaka, BGD; 4 Cardiology, National Institute of Neurosciences and Hospital, Dhaka, BGD; 5 Pediatric Neurology, National Institute of Neurosciences and Hospital, Dhaka, BGD; 6 Clinical Neurology, Bangabandhu Sheikh Mujib Medical University, Dhaka, BGD; 7 Interventional Neurology, National Institute of Neurosciences and Hospital, Dhaka, BGD

**Keywords:** kaplan-meier survival curves, receiver operating characteristic (roc) analysis, stroke mortality, modified rankin scale (mrs), gcs, nihss (national institutes of health stroke scale), troponin i, acute ischemic stroke (ais)

## Abstract

Objective: To assess if elevated cardiac troponin I (cTnI) serves as a sign of unfavorable functional outcomes in ischemic stroke.

Methods: In this single-center prospective cohort study, 100 consecutive patients admitted with acute ischemic stroke (normal troponin I group n = 52, raised troponin I group n = 48) were included. Hospital mortality was documented in both groups; the remaining patients were followed up to 90 days. Then two groups were compared in terms of unfavorable short-term outcomes (Modified Rankin Scale > 3) and mortality. Multivariate logistic regression was conducted to determine the predictive value of elevated cTnI. The Kaplan-Meier curve was drawn and compared to determine the difference in survival between the two groups. To find out the most probable cut-off level for an unfavorable outcome, a receiver operating characteristic (ROC) analysis was conducted.

Result: A higher frequency of coronary artery disease (p=0.030), higher National Institutes of Health Stroke Scale (NIHSS) (p=0.008) score, and lower Glasgow Coma Scale (GCS) (p=0.002) was observed in raised troponin I group. Even after the exclusion of confounding elevated troponin I was found to be an independent predictor of unfavorable outcomes (adjusted odds ratio, OR 8.25 {95% confidence interval, CI: 2.65-25.75}; p<0.001). The patients with raised troponin I had a significantly lower rate of survival after 90 days (p=0.022). The elevated troponin I was observed to have a significantly high accuracy (p<0.001; area under curve, AUC: 0.768 {moderate accuracy}, 95% CI: 0 .676 to 0.861) in predicting unfavorable outcomes.

Conclusion: Elevated cTnI is independently associated with unfavorable short-term outcomes. It is also associated with a lower rate of survival.

## Introduction

Currently, stroke is the second leading cause of disability and death worldwide [1]. One or more biomarkers, such as D-dimer, CRP, brain natriuretic peptide (BNP), and cardiac troponin I (cTnI), have been proposed to predict the outcome of an ischemic stroke [2,3]. Among them, the cardiac biomarker troponin I had a relatively predictable impact [4]. The prevalence of elevated cTnI was found between 14% to 22% of the patients with acute ischemic stroke [5-7]. Even though cTnI is highly precise for myocardial infarction, it does not always reveal the underlying mechanism. Autonomic overactivity leads to cTnI release from cardiac tissue after acute ischemic stroke. This crucial consideration is essential for understanding and interpreting cTnI in patients with ischemic stroke [8]. 

It has not yet been unequivocally proven that elevated cTnI in stroke patients has any bearing on prognosis. Previous research [7,9,10] revealed an association between elevated cTnI levels and poor stroke outcomes and mortality, but subsequent studies [11,12] were unable to replicate these results after controlling for significant confounding variables.

Overall, the idea of troponin I as a biomarker for monitoring outcomes is appealing but needs further validation. An association of raised troponin I with both hospital mortality and the short-term outcome will improve our understanding of stroke pathophysiology and will help the physicians to counsel the patients and optimize the health care resources from primary to tertiary level of care. Therefore, this study has been designed to estimate the impact of troponin I on hospital mortality and short-term outcomes in ischemic stroke. This study aimed to ascertain the prognostic value of elevated cTnI for unfavorable outcomes. Additionally, the characteristics of the patients and the effect of cTnI on survival were also assessed.

## Materials and methods

Study design and subjects

This was a single-center, prospective observational study in a referral neuroscience institute from July 2022 to June 2023. Patients admitted to the department of neurology with acute stroke (defined as acute stroke event within the last 72 hours). After obtaining the clearance of the Institutional Review Board (IRB/NINS/2022/188), prospectively, all consecutive patients with acute ischemic stroke admitted were examined. If stroke symptoms appeared more than 72 hours before hospital admission or if no cTnI values were available, participants were disqualified from the study. Additionally, patients were also ineligible if there was a recurrent stroke, hemorrhagic transformation, acute or recent (within 3 weeks) myocardial infarction, cardiac failure, renal impairment, or pulmonary embolism. Any form of intervention, for example, intravenous thrombolysis with recombinant tissue plasminogen activator (rTPA), mechanical thrombectomy, and decompressive craniectomy were also excluded as they have a proven positive impact on the outcome and may cloud the true outcome values.

Medical history included hypertension, dyslipidemia, diabetes mellitus, a history of stroke, ischemic heart disease, atrial fibrillation, and the presence of renal impairment. After admission, the cTnI of the subjects was immediately (within 72 hours of the onset of symptoms) measured by the hospital lab, and no external report was accepted. For the quantitative analysis of serum cTnI, ARCHITECT STAT by Abbott Diagnostics, Ireland, was utilized. It uses chemiluminescent microparticle immunoassay (CMIA). Troponin I levels of more than 0.034 ng/ml in men and more than 0.016 ng/ml in women were regarded as elevated.

Study procedure

After the selection of the subjects, the nature, purpose, and benefits of the study were explained to each subject or his/her attendant in detail. All subjects were divided into two groups, the normal troponin I group, and the elevated troponin I group. When an adequate number of participants were included in a group, recruitment was continued only in the remaining group. Initially, patients were assessed with the National Institutes of Health Stroke Scale (NIHSS) and Glasgow Coma Scale (GCS) on admission. The NIHSS and GCS both have an established association with the functional outcome of stroke. During the hospital stay, if the patient passed away, mortality was noted. After the clinical improvement or stabilization, patients were discharged either to their homes or a nursing home. These patients were followed up after 90 days by the Modified Rankin Scale (mRS). Follow-ups were done by meeting with the patient face to face but in case a meeting was not possible in time, it was taken from the patient or patient’s relative in person or over the phone or video call. Two groups were compared in terms of outcome. 

Outcome parameters

The functional assessment was done by mRS which measures, disability/dependence and death (score 6) after a stroke. mRS score was obtained during hospital admission, at discharge, and after 90 days. An mRS score of >3 (4-6) was considered an unfavorable outcome [13].

Statistical analysis

Statistical Package for Social Sciences (SPSS), version 22.0 (IBM Corp., Armonk, NY), was used for all the statistical analyses. The normality test (Kolmogorov-Smirnov) revealed how the data were distributed. Then, the normally distributed data were compared by independent t-test. To compare qualitative data between the groups, a Chi-Square test was used. To determine the predictive value of cTnI for the unfavorable short-term outcome, a multivariable logistic regression analysis was conducted. To ascertain the difference in survival between the high cTnI group and the normal cTnI group, Kaplan-Meier curves, and the log-rank test were put to the test. Finally, the accuracy of the cTnI and the best probable cut-off of cTnI for predicting unfavorable outcomes were also determined using a receiver operating characteristics (ROC) curve. At p<0.05, the statistical significance was acknowledged.

## Results

Study population

**Figure 1:**
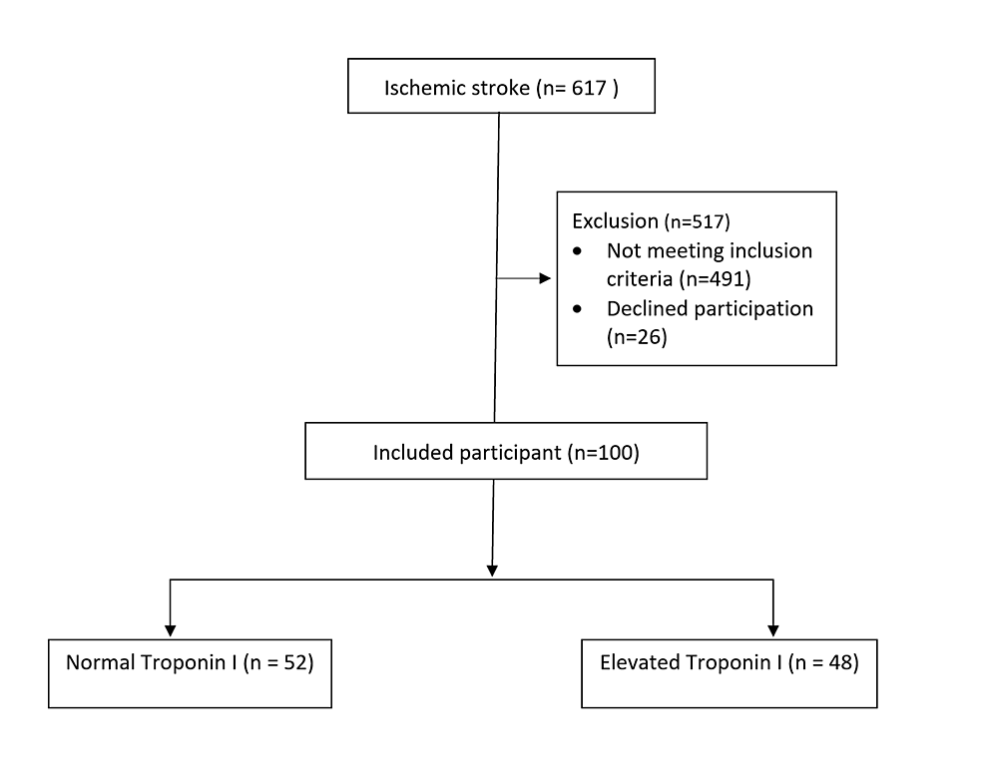
Inclusion method flow chart in the study population.

Between July 2022 and June 2023, 617 patients admitted with the diagnosis of ischemic stroke were screened for this study. According to inclusion and exclusion criteria, a total of 100 acute ischemic stroke patients were included in this study. Among those participants, 52 of them had normal troponin I levels, while 48 of the subjects had high troponin I (Figure 1).

Patient characteristics

The comparison between the baseline characteristics of the study subjects is shown in Table 1. The participants with elevated troponin I, were much older (63.92±14.16 vs 56.50±13.27) compared to participants with normal troponin I but no significant difference was there. The majority of the female (58.3%, p=0.017) patients with acute ischemic stroke had elevated troponin I. Among the risk factors, only ischemic heart disease had a significant (P = 0.030) correlation with elevated troponin I. Among the respondents, the mean NIHSS score was significantly (P = 0.008) higher and GCS was significantly (P = 0.002) lower in patients with elevated troponin I.

**Table 1:** Baseline characteristics of study subjects with and without troponin I (n=100) Data were expressed as frequency, percentages are over column total; p-value was obtained from the Chi-Square test, "a" unpaired t-test; and "*" Fisher's exact test SD = standard deviation, NIHSS = National Institute of Health Stroke Scale, GCS = Glasgow Coma Scale

Variables		All participants N=100	Troponin I		p-value
			Normal (n=52)	Elevated (n=48)	
Age (years)	Mean ± SD^a^	60.06 ± 14.14	56.50±13.27	63.92±14.16	0.080
Gender	Male, n (%)	54 (54%)	34 (65.4%)	20 (41.7%)	0.017
	Female, n (%)	46 (46%)	18 (34.6%)	28 (58.3%)	
NIHSS	Mean ± SD^a^	14.91±5.86	13.42±5.73	16.52±5.63	0.008
GCS	Mean ± SD^a^	10.19±2.18	10.83±1.95	9.5±2.22	0.002
Medical history	Hypertension, n (%)	76 (76%)	39 (75%)	37 (77.1%)	0.810
	Diabetes, n (%)	64 (64%)	38 (73.1%)	26 (54.2%)	0.070
	Dyslipidemia, n (%)	80 (80%)	46 (88.5%)	34 (70.8%)	0.140
	Coronary artery disease, n(%)	22 (22%)	7 (13.5%)	15 (31.3%)	0.030
	Atrial fibrillation, n (%)	11 (11%)	6 (11.5%)	5 (10.4%)	0.860
	Valvular heart disease, n (%)	5(5%)	2 (3.8%)	3 (6.3%)	0.462*
	Smoking habit, n (%)	17 (17%)	12 (23.1%)	5 (10.4%)	0.090
	Family history of stroke, n (%)	20 (20%)	16 (30.8%)	4 (8.3%)	0.050

Outcome parameters

The unfavorable outcome (mRS=4-6) was also significantly higher in participants with elevated troponin I (p<0.001; Table 2). Moreover, after multivariate logistic regression, troponin I was independently associated with (odds ratio: 8.25; 95% CI: 1.18-25.27) increased risk of unfavorable clinical outcomes (Table 2).

**Table 2:** Multivariate logistic regression model for the unfavorable short-term outcome (n=100) CI = confidence interval, NIHSS = National Institute of Health Stroke Scale, GCS = Glasgow Coma Scale, OR = odds ratio

Parameter	Univariate		Multivariate	
	OR (95% CI)	p-value	OR (95% CI)	p-value
Age	1.049(1.02-1.08)	0.004	1.35(0.91-2.00)	0.131
Gender(male)	1.067(0.47-2.45)	0.879		
NIHSS	1.170(1.07-1.27)	<0.001	1.13(1.03-1.26)	0.012
GCS	0.681(0.53-0.87)	0.002	0.87(0.61-1.24)	0.435
Hypertension	1.548(0.60-3.98)	0.365		
Diabetes	1.467(0.62-3.56)	0.382		
Dyslipidemia	0.799(0.25-2.53)	0.703		
Ischemic heart disease	1.256(0.44-3.63)	0.673		
Atrial fibrillation	0.890(0.24-3.81)	0.861		
Troponin I	10.84(3.69-31.79)	<0.001	8.14 (2.63-25.19)	<0.001

**Figure 2:**
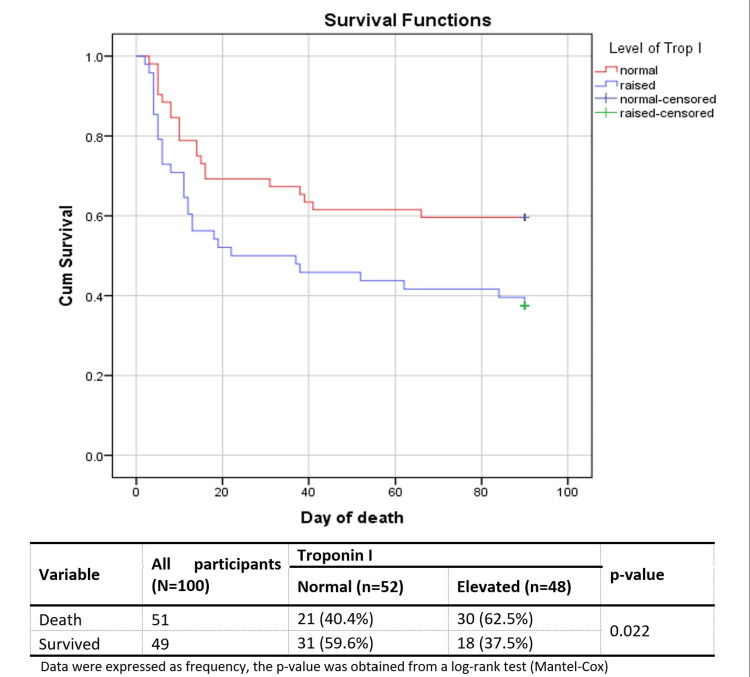
Kaplan-Meier survival curve from day 0 until day 90 showing cumulative survival of ischemic stroke patients with elevated and normal troponin I (N=100)

After 90 days, 49% of the participants survived, and the majority of the mortality was in the elevated troponin I group (62.5% vs. 40.4%). The Kaplan-Meier curves show that there was a distinctive difference in survival between the two groups (p=0.022, Figure 2).

**Figure 3:**
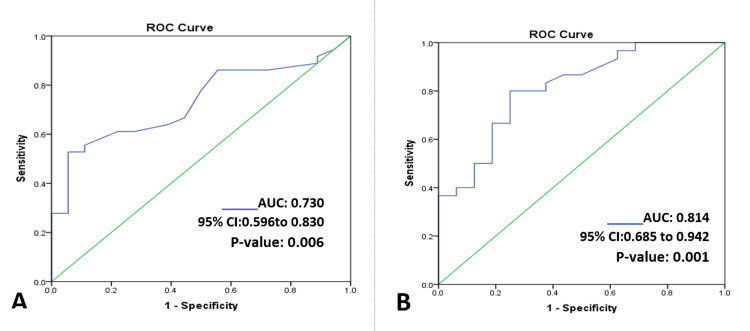
Receiver operating characteristic (ROC) curve showing serum troponin I for the prediction of poor outcome (N=100); (A) in males (n=54), and (B) in females (n=46) AUC = area under curve, CI = confidence interval

The ROC curve was distinctly above the 45^o^ line in both genders. The AUC was 0.730 for males (Figure 3A) and 0.814 for females (Figure 3B). This indicates that elevated serum troponin I is a significant predictor of poor outcomes after 90 days irrespective of gender difference. The best probable cut-off in females 0.016 µg/L has a high sensitivity (80%) and specificity (75%). On the other hand, the best probable cut-off in males 0.033 µg/L has moderate sensitivity(52.8%) but high specificity (94.4%).

## Discussion

The rise in troponin I in acute ischemic stroke may be either due to cardiac dysfunction causing secondary cerebral ischemia or ischemic stroke causing secondary cardiac dysfunction [11,12,14]. Additionally, investigations have hinted that this occurrence can be used as a prognostic indicator to forecast their outcome [10,15]. For this purpose, a prospective cohort study on 100 acute ischemic stroke patients was conducted. After adjusting a broad spectrum of confounding factors our study revealed a significant association between raised troponin I level with unfavorable short-term outcomes and mortality. The best cut-off point for predicting an unfavorable short-term outcome was 0.014 ng/ml.

A large number of the participants in this study had preexisting ischemic heart disease (p=0.030). This is most likely because the ischemic heart mostly goes through remodeling and these remodeled hearts are more vulnerable to injury than healthy ones [10]. Several previous studies also got similar findings [10,16].

The mean NIHSS score was significantly (p=0.008) higher and the mean GCS was significantly lower (p=0.002) in participants with elevated troponin I. Formerly, quite a few studies found significantly higher NIHSS scores in elevated troponin I than in the normal troponin I group [10,15,17]. Contrary to the results of this study, some studies found no significant difference in the mean NIHSS score between the two groups [9].

Later, when the short-term outcome of both groups was compared, the unfavorable outcome (mRS 4-6) among participants with elevated troponin I was significantly (p<0.001) higher than the participants with normal troponin I. Many studies also observed significantly unfavorable outcomes associated with raised troponin I [7,9,10]. This relationship was comparable to other studies of a similar nature [7,9,10,18].

Survival analysis showed a majority (59.6%) of the patients with normal troponin I had an event-free survival over the study period of up to 90 days. On the contrary, the mortality was significantly (p=0.022) higher in the elevated troponin I group. It was concordant with the observation of a few other studies [15,19].

In order to estimate the prognostic accuracy of troponin I a ROC curve was drawn. The ROC curve concluded that cTnI can almost accurately (~72%) predict the unfavorable outcome in acute ischemic stroke. However, the cut-off level (0.014 µg/L) was much lower compared to the cardiac cut (0.04 µg/L) for myocardial infarction [20].

There were several limitations of this study even though every effort was made. As randomization could not be done, bias might have affected the findings. The follow-up time for the study was also brief. The fluctuation in troponin levels throughout time could not be accounted for since only one baseline troponin I level was obtained.

All the patients were hospitalized and received standard treatment according to the hospital protocol.

## Conclusions

Finally, the findings of this study reveal that a raised troponin I level is an independent predictor of unfavorable short-term outcomes in acute ischemic stroke. Moreover, there is also an association with increased mortality in these patients. This study shows that even mild elevation in troponin I is sufficient to predict the unfavorable outcome contrary to cardiac conditions where higher levels are necessary for diagnosis. This provides a new window to plan the diagnosis and management.
